# Exploring accommodations along the education to employment pathway for deaf and hard of hearing healthcare professionals

**DOI:** 10.1186/s12909-022-03403-w

**Published:** 2022-05-06

**Authors:** C. J. Moreland, L. M. Meeks, M. Nahid, K. Panzer, T. L. Fancher

**Affiliations:** 1 Department of Internal Medicine, Dell Medical School at the University of Texas at Austin, 1601 Trinity St, Bldg B, Austin, TX 78712 USA; 2 Center for a Diverse Healthcare Workforce, University of California, Davis, School of Medicine, Sacramento, CA USA; 3 Department of Family Medicine, University of Michigan Medical School, 1018 Fuller St., Ann Arbor, MI 48104-1213 USA; 4 General & Internal Medicine, Weil Cornell Medicine, 420 E 70th St., New York, NY 10021 USA; 5 Department of Internal Medicine, UC Davis School of Medicine, 4610 X Street, #4101, Sacramento, CA 95817 USA

**Keywords:** Disability, Diversity, Deafness, Medical education

## Abstract

**Background:**

Deaf and hard of hearing (DHH) people are an underserved population and underrepresented among healthcare professionals. A major barrier to success for DHH healthcare professionals is obtaining effective accommodations during education and employment. Our objective: describe DHH individuals’ experiences with accommodations in healthcare education.

**Methods:**

We used an online survey and multipronged snowball sampling to recruit participants who identify as DHH and who had applied to a U.S. health professional school (regardless of acceptance status). One hundred forty-eight individuals representing multiple professions responded; 51 had completed their training. Over 80% had been accepted to, were currently enrolled, or had completed health professions schools or residency programs, and/or were employed. The survey included questions addressing experiences applying to health professions programs and employment; satisfaction with accommodations in school and training; having worked with a disability resource professional (DRP); and depression screening.

**Results:**

Use and type of accommodation varied widely. While in school, respondents reported spending a mean of 2.1 h weekly managing their accommodations. Only 50% were highly satisfied with the accommodations provided by their programs. Use of disability resource providers (DRPs) for accommodations was highest during school (56%) and less frequent during post-graduate training (20%) and employment (14%). Respondents who transitioned directly from school to employment (versus via additional training) were more satisfied with their accommodations during school and were more likely to find employment (*p* = 0.02). Seventeen respondents screened positive for risk of depression; a positive screen was statistically associated with lower school accommodation satisfaction.

**Conclusions:**

DHH people study and practice across many health professions. While respondents were mostly successful in entering health professions programs, accommodation experiences and satisfaction varied. Satisfaction with accommodations was related to successful employment and wellness. Low satisfaction was associated with higher likelihood of depression symptoms. To increase representation in the workforce, healthcare professional schools, training programs, and employers should enhance support for the learning and working climates for people with disabilities.

**Supplementary Information:**

The online version contains supplementary material available at 10.1186/s12909-022-03403-w.

## Background

### Barriers for DHH people

Deaf and hard of hearing (DHH) people, who represent about 17% of the total United States (U.S.) population, are an under-recognized medically underserved population [1]. People who are DHH experience significant communication-based barriers to health care, impeding access to medical, oral, mental, and dental health services [ 2–5]. These barriers can lead to negative health and health care outcomes including more emergency department visits, higher risk for depression and food insecurity, and lower knowledge about healthcare conditions and associated risks, including cancer, cardiovascular disease, HIV, and human papillomavirus [6–14]. These communication-related disparities were heightened during the COVID-19 pandemic and universal masking, rendering many hospitals less able to effectively address the needs of their DHH patients [15].

Of note, the general population of people with hearing loss is widely heterogeneous in terms of hearing loss etiology and severity; those who have congenital or early-onset hearing loss are more likely to face challenges of language acquisition and development than those with late-onset hearing loss, who in turn may seek different forms of accommodation than those in the first group. This diversity within the community holds true for communication modalities (some may prefer spoken or signed languages, or both) and cultural identities, with identities including being hard of hearing, deaf, or even Deaf – an identify which, when capitalized, generally refers to those for whom American Sign Language – ASL – or another signed language is central to identity and community. In our paper, we adopt a common convention of describing the community as deaf or hard of hearing, while recognizing that many individuals or even groups identify using other terms. Members of the disability community also use both person-first and disability-first language; to honor both preferences, we use both in this article.

### Concordant and culturally informed care

Effective communication with healthcare professionals is a fundamental need and right for all people, including those who are DHH. Patients who are DHH benefit from working with language-concordant primary care clinicians [16]. Language concordant and culturally informed care provided by DHH healthcare professionals may directly and indirectly impact how providers communicate with and care for patients who are DHH through several mechanisms, including direct communication with patients in signed language (language concordance), education of colleagues who are not DHH in how to serve such patients, and role modeling of appropriate communication strategies for colleagues and educators [17, 18]. Additionally, disabled healthcare students with sensory and physical disabilities are more likely to enter primary care and more likely to intend to care for people with disabilities, including hearing loss [19, 20]. Taken together, providers who are DHH can serve as one mechanism for reducing health care disparities through service to patients with and without disabilities. Despite the aforementioned benefits to reduce health disparities for the DHH population, and the potential for DHH clinicians to mitigate barriers and improve outcomes for DHH patients, people who are DHH continue to be underrepresented as healthcare professionals.

### Barriers to healthcare professions education

There are several identified barriers for students with disabilities across health professions education [21]. Disability resource providers (DRPs, specialists in determining accommodations for educational settings) with limited healthcare profession experience resulting in ill-informed decision-making and potential under-accommodation [22]; lack of clear policies and procedures directing students with disabilities on how to disclose disabilities and request accommodations [21]; and lack of knowledge about assistive technology are among the largest barriers to medical students [21, 22], along with well-described structural barriers in medical schools’ technical standards, admissions practices, and residency programs’ policies [23–29]. Lack of disability access also impacts the mental health of disabled trainees. In a 2021 study, resident physicians who lacked disability-related access to their programs were more likely to experience increases in depressive symptoms [30]. In this same study, no differences were found on measures of mental health for interns that had disability-related access, underscoring the critical nature of effective accommodation and informed decision-making. Systematic barriers have consequences on performance and progression of disabled trainees as well. Another 2021 study showed that medical students with physical and sensory disabilities had higher probabilities of taking a leave of absence than controls, and take longer time to graduation than their non-disabled peers. It has been proposed that the time spent organizing, implementing, and refining accommodations in the clinical setting may partially explain these delays [31].

The majority of studies to date focus on the experiences of DHH learners (i.e., students and trainees) as medical students and physicians, narrowing our understanding of the barriers to the inclusion of learners who are DHH across the broader health professions workforce. If we are to fully realize inclusion of DHH healthcare workforce we must first understand the experiences, including the barriers to education and employment, for all health professions education.

To our knowledge, no database or literature fully describes the accommodation experiences of DHH individuals who aspire to or are engaged in the health professions. This study aimed to 1) identify and describe the accommodation planning, implementation, and satisfaction of individuals who are DHH across a range of health professions programs, practices, and specialties; 2) explore the self-reported mental health of participants; and 3) explore DHH healthcare students’ and professionals’ educational and employment attainment. Our goal in exploring these aims was to describe the demographics of, and accommodations used, by healthcare professionals and students who are DHH. We additionally hypothesized that DHH healthcare students’ and professionals’ effective use of accommodations, as measured by accommodation satisfaction, would be associated with success in obtaining employment and measures of risk of depression.

## Methods

We designed and disseminated an online survey (see Additional file 1) via Qualtrics, beginning April 2019. Given the diversity of communication preferences among DHH healthcare professionals (including signed and/or spoken language use), and that nearly all DHH respondents to a previous survey expressed comfort with English [32], we elected to structure this survey using written English. Healthcare professionals and students in our professional networks received an email invitation to take the survey and to forward the survey to others who might qualify. We also sent the survey to organizations engaged with professionals with disabilities (the Association for Medical Professionals with Hearing Loss [33], the Coalition for Disability in Health Science Education [34], the Society for Physicians with Disability [35]) which in turn emailed the invitation to members and shared it on social media. We likewise posted on our professional social media accounts. Repeat reminders were sent weekly for 7 weeks; the survey closed in June 2019.

Survey questions explored demographics; applications to school, residency, fellowship, or employment after completing training; accommodation utilization and satisfaction; a 2-item depression screening; engagement with a DRP during training; current or planned subspecialty (if any); and current or future plans to serve DHH patients. Questions focused on patients who are DHH, accommodations, and accommodation satisfaction were drawn from a previous survey [32]. The novel question regarding engaging the assistance of a DRP to determine and facilitate accommodations was developed and reviewed by team members, including a DHH physician (CM) and a health professions education DRP (LM). Depression screening was measured via a PHQ-2 questionnaire, a 2-item screening addressing frequency of depressed mood and anhedonia over the past 2 weeks. PHQ-2 scores of 3 or greater indicate a higher current risk of depression [36]. All questions were closed-ended except for numeric responses.

The first two survey questions addressed whether potential participants self-identified as DHH or as having a hearing loss, and whether they had ever applied to a U.S. healthcare professional school (limited to the U.S. due to variation in training curricula among countries). Participants who responded yes to both questions were prompted to continue the survey. The survey began with a consent statement; participant survey initiation constituted consent.

We analyzed responses using descriptive statistics. Not all respondents answered all questions; we report the number of respondents for each question. Association analyses employed chi-squared (for depression screening and accommodation satisfaction in school) or two-sided Fisher’s Exact tests (for accommodation satisfaction and success in obtaining employment), as appropriate. We selected a *p*-value of 0.05 as the significance threshold.

This study was approved as exempt by the institutional review boards at UT Health San Antonio, the University of Michigan Medical School, Ann Arbor, and University of California, Davis Health.

## Results

### Respondents

One hundred and forty-eight DHH respondents across multiple health professions and groups underrepresented in medicine participated in the overall survey (Table 1). The mean age was 33.7 years (range 19–67, median 32). Sixty-eight (47.6%) reported being DHH since birth. Most respondents (73%) self-identified as female; 33% identified as non-white or Hispanic/Latinx and 80.3% identified as white. Fewer than one-third of respondents were immigrants or children of immigrants, first to attend college, or LGBTQ. Regarding language use, 103 (99.1%) reported being comfortable with communicating in English and 1 (0.96%) was uncomfortable; when asked about comfort with signed communication, 34 reported being comfortable, 13 neutral, and 57 uncomfortable. Twelve applied to, and did not begin, health professional school.

**Table 1:** Demographics of 148 deaf and hard of hearing health professional and student survey respondents^a^

Demographic variable	N	(%)
**Sex**		
Female	76	73.0
Male	27	26.0
non-binary	1	1.0
**Race**		
White	83	80.6
Asian or Asian-American	14	13.6
Black, African-American, Hawaiian or other Pacific Islander, American Indian or Alaska Native	7	6.8
Other	7	6.8
Preferred not to answer	2	0.9
**Hispanic or Latinx origin**		
Yes	6	5.8
No	96	92.3
Preferred not to answer	2	1.9
**Membership in other groups underrepresented in healthcare**		
Lesbian, gay, bisexual, transgender, queer	17	17.0
First in your family to attend college	12	12.0
Immigrant or child of immigrant parents	25	25.0
Veteran	0	0.0
None of the above	56	56.0
**Self-identification of hearing loss**		
Deaf or deaf	77	52.7
Hard of hearing	72	49.3
Hearing-impaired	50	34.3
With a hearing loss	41	28.1
**Severity of hearing loss (self-reported)**		
Mild	3	2.1
Moderate	28	19.4
Severe	40	27.8
Profound	71	49.3
Unsure or do not know	2	1.4
**Current level of training or practice**		
Student in health professional school	47	37.6
Residency or fellowship	13	10.4
Graduated school & completed clinical training	51	40.8
No longer in school, did not graduate	2	1.6
Did not begin health professional school	12	9.6
**Current year at school for those who are in school (*n*** **= 44)**		
Year: First	12	27.3
Second	6	13.6
Third	11	25.0
Fourth	7	15.9
Fifth	0	0
Other	8	18.2

^a^Respondents could choose more than one answer

Fifty-two (46.0%) reported current employment in healthcare, with a mean of 5.7 years in their current location (0–30, median 3, SD 5.89). Of those currently employed, 50 described their practice type: solo (3%), a practice with fewer than 10 professionals (7%) or over 10 professionals (9%), group/staff health maintenance organization model (2%), academic (8%), no patient care (2%), and other (19%).

One hundred and one respondents described their current or intended profession (Table 2). Over half represented physician and nursing professions, with wide representation across other professions.

**Table 2:** The current or intended healthcare professions of 105 respondents (including students and graduates)

Specialty	n	(%)
Medicine (Physicians)	36	34.3
Nursing (Nurse and Nurse Practitioner)	25	23.8
Audiology	17	16.2
Veterinary Medicine	6	5.7
Pharmacy	5	4.8
Physician Assistant	5	4.8
Dentistry	4	3.8
Physical Therapy	2	1.9
Occupational Therapy	2	1.9
Clinical Psychologist	2	1.9
Optometry	1	1.0
**Total**	105	100

### Outcome of applications to school and residency

Of 125 respondents who provided information about the application process in the U.S., Table 3 lists reported final outcomes.

**Table 3:** Self-reported final outcomes of 125 respondents’ applications to healthcare professional school, residency, and employment

Final application outcomes	n	(%)
**School**		
Accepted/admitted to program	100	80.0
Wait-listed	1	0.8
Application rejected	7	5.6
Admitted; school subsequently rescinded acceptance	1	0.8
Other	16	12.8
Total	125	100
**Residency or fellowship**		
Accepted or matched	28	87.5
Was not accepted or did not match	3	9.4
Accepted off wait list	0	0.0
Matched on scramble	1	3.1
Accepted to residency/fellowship outside match program	0	0.0
Total	32	100
**Employment**		
Offered a position; respondent accepted	49	80.3
Offered a position; respondent did not accept	0	0.0
Respondent was not offered a position	6	9.8
No further contact after application(s)	2	3.3
Other	4	6.6
Total	61	100

### Accommodations

Respondents’ use of accommodations varied widely by type and overall trends, with more respondents reporting accommodation use in school and fewer respondents reporting accommodations in residency or employment (Table 4). For example, in aggregate, interpreters were less frequently used than other accommodations, yet were more consistently used across all settings. Accommodations are listed by setting (Table 4, including lectures, group discussions, clinical work, and conferences), and stage (school, residency, employment); responses indicated variable use across all settings and stages.

**Table 4:** Types of accommodation used at each stage of education and practice (*n* = 111)^a^

***Accommodation type***	***School***	***Residency***	***Employment***
None	70	15	38
Simple amplified stethoscope	39	6	13
Computer-assisted real-time (CART) captioning	39	4	7
Note-taking services in didactic settings	37	0	13
Sign language interpretation	29	5	8
Stethoscope modified	28	5	13
Modified surgical mask or equivalent substitution	9	2	4
Oral interpretation	5	0	2

^a^Respondents could select more than one accommodation and more than one stage, so responses do not total 111

With regard to time spent planning accommodations, in aggregate, more time was spent in planning accommodations in school when compared to residency, and more in residency than employment. For school-based accommodations, 98 respondents reported spending on average 2.1 h per week planning and navigating accommodations (range 0–30, median 1). For residency, 22 respondents reported 0.9 h on average planning their accommodations (range 0–5, median 0). Time spent on employment-based accommodation planning was even lower with 45 respondents reporting 0.52 h (range 0–8, median 0) navigating workplace accommodations.

### Accommodation satisfaction

For accommodation satisfaction, 101 respondents answered survey questions. Regarding accommodations in school, 51 (50%) reported that their accommodations experience met their needs “extremely well” or “very well,” while 28 (28%) reported being less satisfied with “slightly well” or “not satisfied;” 22% were neutral.

Of 25 respondents who trained in residency or fellowship, 13 (52%) reported being “extremely well” or “very well” satisfied, while 4 (16%) were “slightly” or “not satisfied.” The remaining 32% were neutral.

Among those who transitioned directly from school to employment, accommodation satisfaction was associated with success in obtaining employment (*p* = 0.022). The association between school satisfaction and success in obtaining employment was not statistically significant for those who entered residency between school and employment (*p* = 1.00).

We asked participants whether they worked with a DRP or disability office to assist with coordinating accommodations at each level of training or practice. Of 104 respondents, 58 (56%) reported using a DRP to assist with accommodations during school. Five of 25 (20%) of respondents utilized a similar office or professional in residency, and 6 of 43 (14%) in employment. We found no significant association between use of a DRP and accommodation satisfaction (*p* = 0.216).

### Respondents’ current or planned specialty of practice and patients served

One hundred and five out of 150 respondents (70%) reported their current or planned healthcare specialty; 54 (51%) were categorized as primary or generalist care (Table 5), representing 22.2% of 36 physician and medical student respondents and 32% of 25 nurse and nurse practitioner respondents. Six stated their current or future plans were unknown; of those, 5 reported graduating from their professional school, while 1 was still a student at the time of the survey.

**Table 5:** DHH health professionals and students categorized by current or planned future practice type (primary care /generalist practice vs. subspecialty practice)

	Primary care or generalist practice	Specialty practice	Undecided or not applicable	Total
**Physicians**	8	27	1	36
**Nursing (nurses and nurse practitioners)**	9	14	2	25
**All other health professionals**	37	7	0	44
**Total**	54	48	3	105

Among respondents in current practice, on average they described 33% (range 0–100, median 10) of their patients as DHH. Those in school or residency training anticipated that, on average, 32% (range 0–100, median 20) of their future patients would be DHH.

### Wellness

One hundred and five respondents completed the PHQ-2 screening questions. Seventeen (16%) screened positive, suggesting that these respondents were at higher current risk for depression; of those 17, 9 (9%) were students, residents, or fellows; 1 was a pre-professional student; and 7 had completed training. Positive screens had a small, yet statistically significant association with lower accommodation satisfaction in school (χ^2^ (1, *N* = 90) = 3.92, *P* = 0.048); this association was not present with lower accommodation satisfaction in residency (*p* = 1.0). PHQ-2 score was not statistically significantly associated with profession (trichotomized as physician, nursing, and other profession).

## Discussion

Existing healthcare disparities have been unmasked and exacerbated during the COVID-19 pandemic, which has also emphasized for many the benefits of retaining diverse clinicians [15]. In accordance, our study describes an under-recognized segment of the healthcare workforce: deaf and hard of hearing individuals and their experiences applying to and during health professional schools and training with accommodations. Building on a previous study of medical students and physicians [32], this study broadens the scope of inquiry to the larger DHH workforce by including other healthcare professionals, with nursing professionals representing about one-fourth of our respondents and physicians one-third. Interestingly, audiologists represented the third largest group, consistent with our anecdotal observations of DHH people becoming increasingly interested in hearing health. Respondents were mostly white, female, comfortable with English, with a severe to profound hearing loss. They varied by self-identification as well as comfort with signed communication, and included representation from minoritized groups (such as those who were LGBTQ, or immigrants or children of immigrants), reflecting diversity within the community of those with a hearing loss.

Most respondents were admitted to a health professions program, accepted into post-graduate training, and went on to full employment. While this trend is cautiously promising, more than one in ten respondents reported not being accepted to school or gaining employment, which we suspect is an underestimate since people who were not accepted may have exited the health professions, thus being less likely to be recruited into our survey, or may be reluctant to report this information. Importantly, accommodation satisfaction was associated with gaining employment for those moving directly from school to employment. It may be that students who are appropriately accommodated have more success and confidence in their abilities and as a result are better equipped to engage in accommodation-related discussions while seeking employment; there is also the possibility that more effective accommodations improve the quality of education and training, with subsequent positive effects on candidacy for successful entry into the workforce. Despite our findings of success, work remains to optimize DHH students’ capacity to successfully apply to healthcare professional schools.

Consistent with previous findings, there was wide variation in accommodation need, satisfaction, and utilization of accommodations, and in engagement with a DRP, affirming that DHH people cannot be addressed as a uniform population [37]. Only about half of participants reported satisfaction with accommodations, suggesting that some accommodations may not meet the needs of individuals who are DHH in school or postgraduate training. Additionally, accommodation use may be a burden for some students. For example, while in school, participants invested a mean of 2 personal hours weekly to manage their accommodations (range 0–30 h), raising concerns that DHH students may be burdened with additional administrative duties beyond the already taxing role of navigating professional studies. Notably, our finding parallels those from a study conducted nearly a decade ago, in which respondents reported a mean weekly time investment of 1.3 h [32]. New to this study was the inquiry about the use of a DRP to assist with the accommodations process. Approximately half of respondents reported working with a DRP in school. The use of a DRP to assist with accommodation management dwindled in post graduate education and into employment. The lack of a specialized DRP who understands the health professions environment is a noted barrier to success in the AAMC report on disability in medicine [21]. It may be that the lack of specialization in accommodations in clinical settings, and subsequent mismatch between accommodation need and approved accommodations, is driving the lack of statistical association between DRP use and accommodation experience as identified in our analyses.

Most participants reported an intention to work or are currently working in primary or generalist care (including nurses, pharmacists, and other HCP in addition to physicians, also reflected in specialty choices within professions as reported in Table 5), a lower percentage than reported in the previous study [32]. This difference may be reflective of the smaller number of physician participants in this study, compared to previous investigations focusing solely on physicians. Despite this decline, our findings support recent scholarship on the association between students with sensory/physical disabilities and match to primary care [19].

To our knowledge, no data exist on the number of physicians who serve the DHH population; however, respondents here report that approximately one third of their patients are, or will be, DHH. This estimate is double that of the general population of patients who are DHH (15%) [38], suggesting that DHH health care providers may fill an unmet need for patient communication concordance in the broader DHH population. Previous studies suggest that clinician-patient race and language concordance have the potential to reduce barriers in access to care, improve patient care and adherence, and reduce healthcare disparities [37, 39]. It is possible that these same benefits could occur in DHH physician-patient concordant care. This study did not address whether clinicians and students who are DHH have an interest in working with other minoritized patient populations, though this question should be explored in future work.

Another concern warranting further exploration is the potential current risk for depression among some respondents. In the 2013 study only 2 participants screened as at-risk for depression, while the current study identified 17 participants at risk; though this was not specific to one particular profession. Interestingly, we found a small and statistically significant association between positive depression screening and lower accommodation satisfaction for those in school, a finding that has not been explored thus far in the literature to our knowledge. While our analyses were not designed to investigate causality, it is feasible that dissatisfaction with accommodations or the need to invest significant time in their management could contribute to concerns about the risk of depression.

Taken together, these findings implore healthcare educational institutions to provide focused support for healthcare professional students with disabilities in the form of disability resource expertise, evaluation of accommodation efficacy informed by the learner, and a devoted disability resource professional to facilitate accommodations, relieving students of that duty and allowing them to devote their time to education and training as future healthcare practitioners. By providing high-quality effective accommodations, specific to the learner, healthcare professional programs can enhance the educational pathway to a diverse workforce by recruiting, supporting, and graduating learners with disabilities. Recommendations to this end have already been published for medical schools’ technical standards and residency programs’ disability policies, yet are disproportionately implemented [24–29]. Additionally, in describing the DHH workforce, and their use of accommodations, preclinical students that are DHH may realize a pathway to health professions that has not been well described or investigated.

These results must be interpreted in light of their limitations. As an online survey delivered among professional networks, self-selection and decisions not to respond to all questions impact our ability to generalize each response to the full population of DHH people in healthcare. Sampling bias may also account for the high proportion of female respondents. As previously noted, we do not yet have longitudinal data from a nationally representative set describing healthcare professionals who are DHH, especially since many acquire a disability later in life after attaining employment; we cannot extrapolate from our results to those with late-onset hearing loss. Those in school or practice were possibly more likely to answer, and our results cannot adequately describe those who were not accepted. The small number of respondents who were in or completed residency or fellowship challenges our ability to describe that career stage within our results. Our methodology precludes incorporation of perspectives from education administrators or employers on accommodations. Similarly, we are unable to verify participants’ reports on their work with patients who are DHH, examine the impact of concordance of communication or deafness on patient care, or describe patients’ or non-DHH professional colleagues’ perspectives on such concordant care.

Our findings support and build on previous results. Further research, including qualitative approaches, is needed to explore the drivers of success for people who are DHH, including accommodations, the types and utilization of DRPs, education quality, and wellness, as educational experiences prior to healthcare professional school. This work should consider the intersectional experiences of people with disabilities [40]. It is also essential that school networks and accrediting organizations add disability items to their demographic collection systems so that DHH people can be identified and described more rigorously beyond our sampling methodology. The AAMC has already added disability items to their second-year and graduating medical student questionnaires.

## Conclusions

DHH healthcare students and professionals are increasingly represented across the workforce. Accommodation satisfaction was associated with obtaining employment and with the current risk of mental health concerns. By incorporating expert DRPs and enhancing accommodation quality, educators and employers can better meet the access needs of its learners and elevate the diversity and wellness of our healthcare workforce, and the quality of concordant care to members of the DHH community.

## Supplementary Information

**Additional file 1.**

## Data Availability

The datasets during/and or analyzed during the current study are available from the corresponding author on reasonable request.

## Abbreviations

AAMC: Association of American Medical Colleges
DHH: deaf and hard of hearing
DRP: Disability resource providers
U.S.: United States
