# Supplementary material for: Exploring accommodations along the education to employment pathway for deaf and hard of hearing healthcare professionals
Source: BMC Med Educ. 2022 May 6;22:345. doi: 10.1186/s12909-022-03403-w (PMC9073820; doi:10.1186/s12909-022-03403-w)
Supplement: Supplementary file 1 — Additional file 1. [file 12909_2022_3403_MOESM1_ESM.docx]

**Additional file 1**

# Survey of Deaf & Hard of Hearing Healthcare Students & Professionals

1. Have you ever applied to a health professional school? (This could include medicine, nursing, physical therapy, occupational therapy, veterinary medicine, dentistry, pharmacy, audiology, physician assistant, optometry, clinical psychology, podiatry, chiropractic practice.)
   1. Yes
   2. No

# Questions regarding hearing status

1. How do you identify: (mark all that apply)

- deaf
- Deaf
- hard of hearing
- hearing-impaired
- with a hearing loss
- with normal hearing (as having no hearing loss or impairment)

1. Would you classify your hearing loss as
   - mild
   - moderate
   - severe
   - profound
   - unsure or do not know
2. At what age did you begin to lose your hearing or become deaf?

- At or around birth
- __ years old
- Unknown

# About your health professional school(s)

1. To how many health professional schools did you apply?
   1. What kind of schools? Check all that apply.
      1. Audiologist
      2. Chiropractor
      3. Clinical psychologist
      4. Dentist
      5. Nurse
      6. Nurse anesthetist
      7. Nurse practitioner
      8. Occupational therapist
      9. Optometrist
      10. Pharmacist
      11. Physical therapist
      12. Physician
      13. Physician Assistant
      14. Podiatrist
      15. Veterinarian
   2. What was the outcome of your application to the school(s)?
      1. Accepted/admitted to program
      2. Wait-listed
      3. Application rejected
      4. Admitted, with subsequent rescinding of acceptance
      5. Other: ____
2. Which best describes your current role? (Select one. If you are a student, resident, or other trainee currently training to become a healthcare professional, please select that profession below.)

- Audiologist
- Chiropractor
- Clinical psychologist
- Dentist
- Nurse
- Nurse anesthetist
- Nurse practitioner
- Occupational therapist
- Optometrist
- Pharmacist
- Physical therapist
- Physician
- Physician Assistant
- Podiatrist
- Veterinarian

1. Are you a student in a health professional school or trainee in a residency program?
   1. Yes, currently in a residency or fellowship program
   2. Yes, currently a student in a health professional school
   3. Where do or did you attend school? _______________________

# Health professional school

1. In what year of school are you?
   1. First
   2. Second
   3. Third
   4. Fourth
   5. Fifth
   6. No longer in school, did not graduate
      1. How many years did you complete?
   7. Graduated
      1. Year graduated: ____
2. What is or was your planned specialty?
   1. _____ (free text)
   2. Unknown
   3. Not applicable – no clinical specialization required or planned

# Residency

1. Did you apply to a residency or fellowship program?
   1. No
   2. Yes
      1. To about how many programs did you apply?
         1. What was the outcome of your application to residency or fellowship?
            1. Accepted/Matched
            2. Was not accepted/Did not match
            3. Accepted off wait list
            4. Matched on scramble
            5. Accepted to residency/fellowship outside match program
2. At what institution(s) or program(s) do or did you train for residency? _______________________
3. In what year of training are you? (For resident and fellow physicians, please identify your current year of postgraduate (PGY) training.)
   1. Select from number 1-7
   2. Completed training
      1. In what year?
   3. No longer in residency or fellowship, and I did not complete training in this program.
      1. How many years of training had you completed?
      2. In what year did you complete your most recent year of training at this location?
4. What is or was your residency or fellowship specialty?
   1. _______________
   2. No specialty (e.g., pharmacy residency)
5. Did you complete another residency or fellowship?
   1. No
   2. Yes
      1. In what specialty? ______________ (free text)
      2. At what institution or program? ___ (free text)
6. If currently in residency, do you plan to pursue a fellowship?
   1. No
   2. Yes
      1. In what field? ___ (free text)

# Current or future employment after training

1. Did you apply for employment in one or more healthcare position(s)?
   1. What was the outcome of your application(s) for employment? (Check all that apply.)
      1. Offered a position, and I did not accept
      2. Was not offered a position
      3. No further contact after application
      4. Other _____ (free text)

*For the following questions, please choose answers that you feel best describes your career since completing your training.*

1. Which of the following best describes your primary practice? Choose one of the following.
   1. Solo practice
   2. Small group practice (2-9 professionals in your specialty)
   3. Large single specialty group (10+ professionals in your specialty)
   4. Large multispecialty group (10+ professionals in your specialty)
   5. Group/staff model HMO
   6. Academic group practice
   7. No patient care
   8. Other: _______________________
2. What year did you start working at your primary work location? _ _ _ _
3. During your practice, how many of your patients would you estimate use:
   1. Spoken English ___ %
   2. ASL or signed English ___ %
   3. Other spoken language ___ %
   4. Other language: _____________

# About your patients

1. [completed training] What percent of your current patients would you estimate are deaf, hard of hearing, or hearing impaired? ___ %
2. [completed training] What percent of your clinical time would you estimate you spend with deaf, hard of hearing, or hearing impaired patients? ___ %
3. [still in school or residency] Do you plan to work with deaf, hard of hearing, or hearing impaired patients after completing school or residency?
4. According to your current plans to practice after school or residency, what percent of your future patients do you expect will be deaf or hard of hearing? ___ %

## About accommodations you have used

1. During school
   1. Computer-assisted real-time (CART) captioning
   2. Sign language interpretation
   3. Oral interpretation
   4. Note-taking services
   5. Simple amplified stethoscope
   6. Stethoscope modified for your use
   7. Modified surgical mask or equivalent substitution for a surgical mask
   8. Other: ___
2. How well did your accommodations satisfy your needs?
   1. (Very well, fairly well, somewhat well, not well, not well at all)
   2. What was each used for? (lectures, clinical rotations, conferences)
3. On average, how many hours per week did YOU personally spend arranging or scheduling these accommodations, as opposed to a disability coordinator or other?
4. During residency and/or fellowship
   1. Computer-assisted real-time (CART) captioning
   2. Sign language interpretation
   3. Oral interpretation
   4. Note-taking services
   5. Simple amplified stethoscope
   6. Stethoscope modified for your use
   7. Modified surgical mask or equivalent substitution for a surgical mask
   8. Other: ___
5. On average, how many hours per week did YOU spend arranging or scheduling these accommodations? ___
6. Were these accommodations arranged by a specialized disability services provider?
7. During current practice after completing training
   1. Computer-assisted real-time (CART) captioning
   2. Sign language interpretation
   3. Oral interpretation
   4. Note-taking services
   5. Simple amplified stethoscope
   6. Stethoscope modified for your use
   7. Modified surgical mask or equivalent substitution for a surgical mask
   8. Other: ___
8. On average, how many hours per week did you spend arranging or scheduling these accommodations? ___
9. I feel that my institution has been supportive in providing the accommodations I need.
   1. Strongly agree
   2. Agree
   3. Neutral
   4. Disagree
   5. Strongly disagree

# Questions about yourself

1. What best describes you?

- Male
- Female
- Gender non-conforming

1. What is your age in years? ______
2. What is your relationship status?

- Married or living as a member of a committed couple
- Divorced or separated
- Widowed
- Single or never married
- Prefer not to answer

1. Are you of Hispanic or Latino origin or descent?

- Yes, Hispanic or Latino
- No, not Hispanic or Latino
- Prefer not to answer

1. How would you describe yourself? Check all that apply.

- White
- Black or African-American
- Asian
- Native Hawaiian or other Pacific Islander
- American Indian or Alaska Native
- Another race (free text) _______
- Prefer not to answer

1. Do you identify as a member of any of the following groups:
   1. Lesbian, gay, bisexual, transgender, queer
   2. First in your family to attend college
   3. Immigrant or child of immigrant parents?
   4. Veteran
2. How comfortable are you using English to communicate?
   1. Very comfortable
   2. Comfortable
   3. Not comfortable
   4. Not comfortable at all
3. How comfortable are you using signed communication (including American Sign Language, signed English, or another visual communication system) to communicate?
   1. Very comfortable
   2. Comfortable
   3. Not comfortable
   4. Not comfortable at all

# Questions about your health

*We would like to ask questions about your health.*

1. In the past two weeks, have you been bothered by:
   1. Little interest or pleasure in doing things?
      1. Not at all
      2. several days
      3. more than half the days
      4. nearly every day
2. Feeling down, depressed, or hopeless?
   - 1. Not at all
     2. several days
     3. more than half the days
     4. nearly every day
3. Do you have any other comments to share with us? If so, please comment below.
   1. ________________________________
